# Predictors of Breastfeeding Duration among Women in Kuwait: Results of a Prospective Cohort Study

**DOI:** 10.3390/nu6020711

**Published:** 2014-02-20

**Authors:** Manal Dashti, Jane A. Scott, Christine A. Edwards, Mona Al-Sughayer

**Affiliations:** 1 Human Nutrition, School of Medicine, College of Medical Veterinary and Life Sciences, University of Glasgow, Glasgow G31 2ER, UK; E-Mails: manaldashti@yahoo.com (M.D.); christine.edwards@glasgow.ac.uk (C.A.E.); 2 Nutrition Unit, Jaber Al-Ahmed Armed Forces Hospital, Ministry of Defense, Sabhan 46004, Kuwait; 3 School of Public Health, Curtin University, Perth 6102, Australia; 4 Department of Biological Sciences, Faculty of Science, Kuwait University, Safat 13060, Kuwait; E-Mail: mona.alsughayer@ku.edu.kw

**Keywords:** breastfeeding, duration, determinants, Middle East

## Abstract

The purposes of this paper are to report the prevalence of breastfeeding to six months among women in Kuwait and to determine the factors that are associated with the duration of breastfeeding. A cohort of 373 women recruited from maternity wards in four hospitals in Kuwait city were followed from birth to 26 weeks postpartum. The association of any and full breastfeeding duration and predictor variables were explored using multivariate Cox’s proportional hazards models. At six months, 39% of all infants were receiving some breast milk and only 2% of infants had been fully breastfed to 26 weeks. Women born in other Arab countries were less likely to discontinue breastfeeding than women born in Kuwait. Other factors positively associated with breastfeeding duration were level of maternal education, higher parity, infant being demand fed in hospital and a preference for breastfeeding on the part of the infant’s father and maternal grandmother. The introduction of a pacifier before four weeks of age and the mother intending to return to work by six months were negatively associated with duration. These findings present a number of opportunities for prolonging breastfeeding duration in Kuwait.

## 1. Introduction

Breastfeeding is an unequalled way to feed an infant. In addition to its unique nutritional properties, human breast milk contains a wide-variety of immunoprotective factors that augment the immature immune system of the infant [1]. Infants who are formula fed are at greater risk of infections common to infancy including gastroenteritis, respiratory infection and otitis media [2]. The World Health Organization (WHO) and the United Nations Children’s Fund (UNICEF) recommend that infants be exclusively breastfed for the first six months of life with breastfeeding continuing for up to two years of age or beyond [3]. The wide-spread practice of delayed initiation of breastfeeding and prelacteal feeding [4,5,6,7,8], along with the early introduction of complementary feeding [9] in the Middle Eastern region however, mean that very few infants in this region are exclusively breastfed from birth for six months as recommended.

Breastfeeding practices are influenced by a complex mix of factors which are related to maternal and family socio-demographic characteristics, biomedical factors, health-care practices, psychosocial factors, social support, community attitudes, and public policy factors [10,11]. The direction of effect of these factors is not consistent across all cultures. For instance, in industrialised countries, better educated women are more likely to initiate breastfeeding and to breastfeed for longer than their less educated counterparts, whereas in poorer countries the opposite tends to be the case. In common with industrialised countries [10,11,12], amongst Middle Eastern women breastfeeding duration has been positively associated with maternal age [13,14,15,16] and parity [14,17,18]. Whereas inconsistent associations have been reported for level of maternal education with breastfeeding duration being associated both negatively [16,19,20] and positively [17] with a higher level of maternal education. Other factors reported to be negatively associated with duration of breastfeeding include maternal employment [17,19,20,21,22], mode of delivery [21,23,24,25] and the use of infant formula while in hospital [24,25,26].

Regular breastfeeding surveillance is essential to determine the extent to which national breastfeeding targets are being met, the impact of breastfeeding promotion interventions and how breastfeeding practices are changing over time. In addition, it is important to investigate the determinants of infant feeding practices so that breastfeeding interventions can be targeted at the most vulnerable population groups and address potentially modifiable risk factors which adversely affect breastfeeding practices. Relatively few studies have investigated infant feeding practices in Kuwait [27,28,29] and none have been longitudinal in nature. The reported mean duration of breastfeeding appears to have declined from 6.4 months in 1988 [27] to 4.9 months in 1997 [28] and there is a lack of more recent data to determine if this downward trend has continued. The aim of the Kuwait Infant Feeding Study (KIFS) was to identify the incidence and prevalence of breastfeeding up to 26 weeks postpartum among a population of women living in Kuwait and to identify the factors associated with the initiation and duration of breastfeeding. The determinants of breastfeeding initiation have been reported previously [30] and the purposes of this paper, therefore, are to report the prevalence of breastfeeding to six months and to determine the factors that are associated with the duration of breastfeeding.

## 2. Experimental Section

### 2.1. Recruitment of Subjects

A prospective cohort study of infant feeding practices among women in Kuwait was conducted between October 2007 and October 2008. The study methods have been described previously [30], but briefly mothers were recruited from three major public hospitals and one private hospital located in Kuwait City. Within 72 h of delivery, eligible mothers were visited and invited to participate in the study by the researcher (MD) who provided a written and verbal description of the study. Women were considered to be eligible for the study if they were able to read or understand Arabic or English and had delivered a live, healthy, singleton of 36 weeks or more gestational age. Mothers whose infants were admitted to the Special Care Nursery (SCN) for minor illnesses or observation were eligible for recruitment.

The study was approved by the Medical Faculty Ethics Committee of the University of Glasgow, (Application No. FM03906: Approved May 29, 2007) and by the Ministry of Health in Kuwait. Participants provided signed informed consent and were advised that they could refuse to participate or withdraw from the study at any time, without prejudicing their post-natal care or the care of their baby.

### 2.2. Data Collection

Mothers who agreed to join the study were interviewed face-to-face to complete a baseline questionnaire prior to discharge from hospital. Women who declined to participate were asked to provide some basic socio-demographic data to determine if the sample were representative of the population of women giving birth at the participating hospitals. All participants were followed up by telephone interview at 6, 12, 18 and 26 weeks postpartum. Data were collected using questionnaires previously used in similarly designed studies of Australian women [31] and modified slightly to meet the needs of this study population. Information on socio-demographic characteristics, maternal lifestyle factors, infant characteristics, biomedical factors, hospital practices, psychosocial factors and feeding practices were collected at baseline. Information on current feeding practices, changes to feeding practices, breastfeeding experiences and introduction of a pacifier were collected during follow-up interviews.

#### Infant Feeding Assessments

Breastfeeding terms used in this study were those defined by the World Health Organization [3]. An infant was considered to be *exclusively breastfed* when he or she had received only breast milk with no other liquids (except for oral rehydration solutions (ORS), drops or syrups) or complementary (solid) foods, and to be *predominantly breastfed* when he or she received breast milk as the main source of nourishment, with certain liquids (water, water-based fluids, fruit juices, ritual fluids, ORS, drops or syrups) but received no other liquids (including formula milk and non-human milks) or complementary foods. *Full breastfeeding* was defined as either exclusive or predominant breastfeeding [32]. *Any breastfeeding* was defined as an infant who was receiving breast milk, with or without formula, other milks, fluids or complementary foods. Duration of exclusive, predominant and any breastfeeding was determined by using information about the age at which other types of milks, liquids (e.g., water, fruit juice) and/or complementary foods were introduced in the first six months of life. In this study, *prelacteal feeding* was defined as the act of giving any liquid or food item (except breast milk) to a newborn within the first three days after birth [33].

### 2.3. Statistical Analysis

We explored the associations of breastfeeding duration and a variety of characteristics and practices reported in the literature to be associated with the duration of breastfeeding using Cox’s proportional hazards model. This model allows joint estimation of the effects of independent variables on the risk of cessation of breastfeeding and can be used to analyze data that contain censored observations [34]. We tested the role of: (1) socio-demographic factors (maternal age, education, country of birth, employment plans for six month postpartum); (2) maternal lifestyle factors (pre-pregnancy BMI calculated from self-reported weight and height, smoking during pregnancy); (3) infant factors (gender, having spent time in the SCN); (4) biomedical factors (parity, delivery method, breastfeeding problems at baseline, breastfeeding problems at six weeks postpartum, age at which pacifier was introduced) (5) hospital practices (time to first breastfeed, composition of infant’s first feed, use of prelacteal feeds, infant roomed-in, infant demand fed in hospital); and (6) psychosocial factors (when decided on feeding method, whether pregnancy was planned, father’s feeding preference, maternal grandmother’s feeding preference, paternal grandmother’s feeding preference). The association of individual variables and the duration of any and full breastfeeding was first evaluated in a univariate model. Any variable with a *P*-value of <0.100 was then included in a multivariate model which was reduced using the backward stepwise procedure. The fitness of each model was assessed at every step to avoid dropping non-significant variables that affected the model fitness. All variables in the final model were variables for which, when excluded, the change in deviance compared with the corresponding statistics on the relevant degrees of freedom was significant. Statistical analyses were performed using the Statistical Package of Social Sciences version 19 (SPSS Inc., Chicago, IL, USA).

## 3. Results

A total of 439 women were invited to participate in the study and 373 mothers completed the baseline questionnaire while in hospital, giving a response rate of 85%. There were no significant differences between participants and those declining to participate (*n* = 66) with respect to age (χ^2^ 4.413, *P* = 0.110), level of education (χ^2^ 2.455, *P* = 0.117) and chosen method of feeding at discharge (χ^2^ 447, *P* = 0.800), suggesting that the sample was representative of the population from which it was drawn [30]. In all, 80 women dropped out of the study prior to completing the final follow-up interview at 26 weeks however, there were no differences in the age, level of education and chosen feeding method of those who completed or withdrew from the study (data not reported). Data for the duration of any and full breastfeeding for women who withdrew were censored in accordance with the woman’s status at the time of last contact, allowing all participants to be included in the survival analysis.

Almost all women (92.5%) initiated breastfeeding, and at six months, just over one third of all infants (39%) were receiving some breast milk and only 2% of infants had been fully breastfed to 26 weeks (Table 1). As not all women had ceased breastfeeding it was not possible to estimate mean duration of breastfeeding, however the median duration of any breastfeeding was 13.9 weeks and the mean duration of those women who had stopped breastfeeding before 26 weeks was six weeks. The median duration of full breastfeeding was less than one week with 50% of infants having received formula feeds within the first week post-partum.

**Table 1:** Prevalence ^a^ of full and any breastfeeding at selected ages.

Age (weeks)	Any Breastfeeding (%)	Full Breastfeeding (%)
4	66	31
8	56	26
12	53	22
26	39	2

^a^ Survival analysis using censored cases.

The majority of women who initiated breastfeeding (*n* = 345) were between 25 and 34 years of age (64.3%), born in Kuwait (54.2%) and had completed 12 or more years of education (78%). Just over one third of women had delivered by Caesarean section (36.5%). Delayed initiation of breastfeeding and prelacteal feeding were the norm amongst this cohort with just over one half of women (52.8%) initiating breastfeeding more than 24 h after giving birth and the majority of infants (88.7%) receiving formula as either their first feed and/or at some time during the first three days of their hospital stay (Table 2). Only one third of women who initiated breastfeeding left hospital fully breastfeeding their infant, with the majority of women (59.4%) partially breastfeeding. A small number of women (*n* = 29, 8.4%) who initiated breastfeeding left hospital exclusively formula feeding.

### 3.1. Univariate Analysis

Due to the small number of breastfed infants (11.3%) who had been exclusively breastfed whilst in hospital we investigated the association of covariates with the duration of full and any breastfeeding for the first six months of life. In the univariate analysis (Table 2), the duration of any and/or full breastfeeding was associated with socio-demographic factors (maternal age, country of birth and level of education), parity, prelacteal feeding, introduction of a pacifier, whether a mother had roomed-in for 24 h with her infant and psychosocial factors (partner’s and maternal grandmother’s support for breastfeeding).

### 3.2. Multivariate Analysis

Table 3 shows the results of the multivariate analysis. After adjustment, maternal level of education and country of birth were independently associated with both duration of full and any breastfeeding. Mothers with 12 or more years of education were less likely to stop any (Adjusted Hazard Ratio (AdjHR) = 0.68) and full (AdjHR = 0.74) breastfeeding during the six month follow-up period compared with mothers with less than 12 years of education. Similarly, mothers born in other Arab countries were less likely to stop any (AdjHR = 0.53) and full (AdjHR = 0.65) breastfeeding compared with women born in Kuwait. Mothers who did not intend to return to work within six months post-partum (AdjHR = 0.76) and those who did not experience breastfeeding problems in hospital (AdjHR = 0.80) were less likely to have stopped full breastfeeding. Conversely, women who did not feed on demand while in hospital (AdjHR = 1.28) or whose partner preferred formula feeding or was ambivalent as to how his child was fed (AdjHR = 1.33) were more likely to stop full breastfeeding. Multiparous women (Adj HR = 0.63) were less likely to cease any breastfeeding while those women who introduced a pacifier to their infant before four weeks (AdjHR = 1.66) or whose own mother preferred formula feeding or was ambivalent as to how her grandchild was fed (AdjHR = 2.11) were more likely to stop breastfeeding during the six month follow-up period.

### 3.3. Reasons for Discontinuing Breastfeeding

The reasons given by mothers for stopping breastfeeding are given in Table 4. The majority of women (86.8%) indicated that they were concerned about the adequacy of their breast milk in terms of either quantity or quality. Almost half (49.1%) indicated that their baby had either weaned them self, preferred a bottle or were ready for solids. A notable proportion stopped breastfeeding because they had returned to work or study. Only a small number of women cited mother-centered reasons like inconvenience and dislike of breastfeeding. The reasons for cessation did not vary markedly according to the infant age at which women stopped breastfeeding.

## 4. Discussion

While breastfeeding initiation is virtually universal amongst women living in Kuwait, targets for breastfeeding duration are not being met, with no woman in this study exclusively breastfeeding to six month of age and only 2% of women fully breastfeeding their infants to this age. As not all women had ceased breastfeeding by 26 weeks, it was not possible to estimate mean duration of breastfeeding however, the median duration of any breastfeeding was slightly more than three months and the mean duration of those women who had stopped breastfeeding before 26 weeks was six weeks. This suggests that the mean duration of any breastfeeding in Kuwait is likely to be less than the 4.9 months reported in 1997 [28] and that breastfeeding duration is declining.

In this study, women born in other Arab countries were less likely to have discontinued any and full breastfeeding than women born in Kuwait or other countries. These women are likely to be the wives of Middle Eastern guest workers employed in the oil and construction industries and the infant feeding practices of these women likely reflect those of their home country where children are breastfed for longer than infants in Kuwait. For instance, contemporaneous studies have reported a mean duration of breastfeeding of 8.6 months in the UAE [16] and 7.6 months in Bahrain [35], and a median duration of 12.4 months in Jordan [36]. A recent study in Kuwait reported that Kuwaiti mothers use bottle feeding more than the non-Kuwaiti mothers [37] which is consistent with our findings. Al Fadli *et al.* [37] proposed that such practice could be explained by the lifestyle changes that occurred in Kuwait due to oil revenue and through using modern technology similar to what happened in Western countries in the 1960s and 1970s.

**Table 2:** Characteristics of a cohort of mother-infant dyads who had breastfed (*n* = 345) and the unadjusted association with the risk of discontinuing any or full breastfeeding during the six months’ follow-up (Crude Hazards Ratio and 95% Confidence Interval).

Characteristic	*N* ^a^	%	Any Breastfeeding			Full Breastfeeding		
			Crude HR	95% CI	*P* value	Crude HR	95% CI	*P* value
***Maternal Factors***								
Age (years)					0.018			0.380
<25	77	22.3	1.36	0.83–2.26		0.95	0.66–1.38	
25–34	222	64.3	0.82	0.52–1.29		0.83	0.61–1.14	
≥35	46	13.3	1.00			1.00		
Mother’s country of birth					<0.001			0.002
Kuwait & Gulf States	187	54.2	1.00			1.00		
Other Arab countries ^b^	119	34.5	0.44	0.31–0.63		0.66	0.52–0.83	
Other non-Arab Countries	39	11.3	0.77	0.48–1.23		0.92	0.65–1.30	
Years of education					0.002			0.090
<12	76	22.0	1.00			1.00		
≥12	269	78.0	0.59	0.42–0.83		0.80	0.62–1.04	
Employment intention for 6 months postpartum					0.156			0.055
Intend to be working	136	39.4	1.00			1.00		
Do not intend to be working or don’t know	209	60.6	0.80	0.59–1.09		0.81	0.65–1.00	
Smoked pregnancy					0.358			0.706
Yes	20	5.8	1.00			1.00		
No	325	94.2	0.73	0.37–1.43		0.92	0.58–1.44	
Pre-pregnancy Body Mass Index (kg/m^2^)					0.623			0.964
<24.99	149	43.2	1.00			1.00		
25.00 to 29.99	124	35.9	1.02	0.72–1.45		1.03	0.81–1.30	
≥30	72	20.9	1.21	0.81–1.82		0.99	0.75–1.31	
***Infant factors***								
Gender					0.962			0.709
Male	176	51.0	1.00			1.00		
Female	169	49.0	0.99	0.73–1.35		1.04	0.84–1.29	
Spent time in Special Care Nursery					0.800			0.148
Yes	61	17.7	1.00			1.00		
No	284	82.3	1.05	0.70–1.58		0.81	0.62–1.08	
***Biomedical factors***								
Parity					0.014			0.081
Primiparous	112	32.5	1.00			1.00		
Multiparous	233	67.5	0.67	0.49–0.92		0.82	0.65–1.03	
Delivery method					0.690			0.723
Vaginal	219	63.5	1.00			1.00		
Caesarean Section	126	36.5	1.07	0.78–1.46		1.04	0.84–1.30	
Breastfeeding problems in hospital					0.100			0.045
Yes	126	36.5	1.00			1.00		
No	219	63.5	0.77	0.56–1.05		0.80	0.64–1.00	
Breastfeeding problems at 6 weeks postpartum					0.165			0.402
Yes	153	44.3	1.00			1.00		
No	192	55.7	1.25	0.91–1.70		1.10	0.89–1.36	
Age pacifier introduced					<0.001			0.244
<4 weeks	113	32.8	1.99	1.44–2.76		1.30	0.96–1.75	
At or after 4 weeks	26	7.5	1.36	0.78–2.37		1.05	0.82–1.35	
Not using a pacifier at 26 weeks	206	59.7	1.00			1.00		
***Hospital practices***								
Time to first breastfeed					0.158			0.424
Within 6 h of delivery	81	23.5	1.37	0.83–2.25		1.18	0.86–1.63	
Between 6 and 24 h	68	19.7	1.50	0.99–2.26		1.18	0.91–1.54	
More than 24 h after delivery	182	52.8	1.00			1.00		
Missing	14	4.1	-			-		
Infant’s first feed					0.162			0.336
Formula/other	277	80.3	1.00			1.00		
Breast milk/colostrum	68	19.7	0.75	0.49–1.13		0.89	0.68–1.15	
Prelacteal feed given					0.008			0.031
Yes	306	88.7	1.00			1.00		
No	39	11.3	0.42	0.22–0.80		0.69	0.50–0.97	
Infant roomed-in for 24 h/day					0.183			0.401
Yes	183	53.0	1.00			1.00		
No	162	47.0	0.81	0.59–1.10		1.10	0.89–1.36	
Infant fed on demand in hospital					0.042			0.064
Yes	245	71.0	1.00			1.00		
No	100	29.0	1.41	1.01–1.95		1.25	0.99–1.58	
***Psycho-social factors***								
When decided how to feed infant					0.884			0.089
Before pregnant	262	75.9	1.00			1.00		
After pregnant	83	24.1	0.97	0.66–1.43		1.24	0.97–1.59	
Planned pregnancy					0.155			0.085
Yes	196	56.8	1.00			1.00		
No	149	43.2	0.80	0.58–1.09		0.83	0.67–1.03	
Father’s infant feeding preferences					0.008			0.007
Prefers breastfeeding	280	81.2	1.00			1.00		
Prefers Bottle or ambivalent	65	18.8	1.64	1.14–2.36		1.46	1.11–1.92	
Maternal grandmother’s infant feeding preference					0.004			0.013
Prefers breastfeeding	312	90.4	1.00			1.00		
Prefers Bottle or ambivalent	33	9.6	2.06	1.26–3.36		1.59	1.10–2.28	
Paternal grandmother’s infant feeding preference					0.448			0.400
Prefers breastfeeding	261	75.7	1.00			1.00		
Prefers Bottle or ambivalent	84	24.3	1.15	0.81–1.63		1.11	0.87–1.42	

^a^ *N* = Number of subjects; ^b.^Other Arab countries included Algeria, Egypt, Iran, Iraq, Jordan, Lebanon, Morocco, Palestine, Qatar, Saudi Arabia, Syria, Yemen.

**Table 3:** Factors independently associated with the risk for discontinuing any or full breastfeeding in a cohort of mother-infant dyads followed to six months postpartum (*n* = 345).

Characteristic	Any Breastfeeding			Full Breastfeeding		
	Adjusted ^a^ HR	95% CI	*P* Value	Adjusted ^b^ HR	95% CI	*P* Value
Mother’s country of birth			0.003			0.001
Kuwait & Gulf States	1.00			1.00		
Other Arab countries ^c^	0.53	0.36–0.76		0.65	0.51–0.83	
Other non-Arab Countries	0.75	0.46–1.22		0.93	0.65–1.33	
Years of education			0.030			0.030
<12	1.00			1.00		
≥12	0.68	0.47–0.96		0.74	0.56–0.97	
Employment intention for 6 months postpartum						0.022
Intend to be working	NS ^d^			1.00		
Do not intend to be working or don't know				0.76	0.60–0.96	
Parity			0.005			
Primiparous	1.00			NS		
Multiparous	0.63	0.46–0.87				
Age pacifier introduced			0.013			
<4 weeks	1.66	1.18–2.33		NS		
At or after 4 weeks	1.25	0.71–2.20				
Not using a pacifier at 26 weeks	1.00					
Prelacteal feed given			0.111			
Yes	1.00			NS		
No	0.59	0.31–1.13				
Breastfeeding problems in hospital						0.046
Yes				1.00		
No	NS			0.80	0.64–0.99	
Demand fed in hospital						0.040
Yes	NS			1.00		
No				1.28	1.01–1.62	
Father’s infant feeding preferences						0.045
Prefers breastfeeding	NS			1.00		
Prefers Bottle or ambivalent				1.33	1.01–1.77	
Maternal grandmother’s infant feeding preference			0.005			
Prefers breastfeeding	1.00			NS		
Prefers Bottle or ambivalent	2.11	1.26–3.54				
	−2 Log Likelihood = 1681.143			−2 Log Likelihood = 3441.922		

^a^ Adjusted for mother’s age, country of birth, level of education, parity, infant received prelacteal feed, age pacifier introduced, father’s and maternal grandmother’s infant feeding preference; ^b^ Adjusted for mother’s country of birth, level of education, employment intention for six months post-partum, parity, breastfeeding problems at baseline, infant received prelacteal feed, infant demand fed in hospital, when infant feeding decision was made, whether pregnancy was planned, father’s and maternal grandmother’s infant feeding preference; ^c^ Other Arab countries included Algeria, Egypt, Iran, Iraq, Jordan, Lebanon, Morocco, Palestine, Qatar, Saudi Arabia, Syria, Yemen. ^d^ NS = non-significant.

**Table 4:** Reasons ^a^ for stopping breastfeeding.

Reason	Stopped < 4 weeks (*N* ^b^ = 51)		Stopped 4–12 weeks (*N* = 68)		Stopped 13–26 weeks (*N* = 40)		Total (*N* = 159)	
	*N*	%	*N*	%	*N*	%	*N*	%
Concerned about quantity and quality of breast milk	42	82.4	59	86.8	37	92.5	138	86.8
Baby weaned self, prefers bottle or ready for solids	24	47.1	31	45.6	23	57.5	78	49.1
Returned to work or study	24	47.1	24	35.3	19	47.5	67	42.1
Breastfeeding too difficult or requires too much motivation	4	7.8	8	11.8	2	5.0	14	8.8
Mother ill, stressed or too tired	3	5.8	4	5.9	3	7.5	10	6.3
Mother-centered reasons (breastfeeding inconvenient, dislike breastfeeding, concern for effect on figure, “done my bit”)	3	5.9	3	4.4	3	7.5	9	5.7
Breast-related problems (cracked or sore nipples, engorgement, mastitis)	1	2.0	1	1.5	2	5.0	4	2.5

^a^ Women may have given more than one reason for stopping; ^b^ *N* = number of subjects.

This study found no independent association with maternal age but, consistent with the findings of studies of women in Western countries [11,12], breastfeeding duration was positively associated with level of maternal education and parity, and negatively associated with maternal employment. Women with 12 or more years of education were less likely to have discontinued any or full breastfeeding compared with women with less than 12 years of education. Multiparous women were less likely to discontinue any breastfeeding than primiparous women which is consistent among women from Western [11,12] and other Middle-Eastern [14,18,22,23] countries. Previous breastfeeding success is a strong predictor of breastfeeding duration [12] and in general women breastfeed for longer with each successive pregnancy.

While there was no association with the duration for any breastfeeding, women who did not intend to return to work within 26 weeks were less likely to have discontinued full breastfeeding than those who planned to return to work. This suggests that women supplement breastfeeding with formula feeding either on return to work or in preparation for a return to work. This negative association between early return to work and breastfeeding duration has been reported in studies of women from other Middle-Eastern countries [17,19,20,22] and is consistent with studies of women from Western countries [31,38,39,40], and suggests that women everywhere have difficulty combining working with exclusive breastfeeding.

Other factors negatively associated with the continuation of full breastfeeding were whether prior to discharge the mother had experienced breastfeeding problems or her infant had been fed on demand, both of which may be inter-related. Milk production is directly related to suckling frequency [41] and there is evidence that fixed feeding schedules lead to insufficient milk supply and breastfeeding problems [42]. These findings highlight the importance of unrestricted breastfeeding in the early post-partum period to the successful establishment of breastfeeding. Hospital staff should encourage demand feeding and support and encourage women to persevere when they are experiencing difficulty establishing breastfeeding rather than resorting quickly to supplementing breast milk with formula.

This was the first study to investigate the association between pacifier use and breastfeeding duration among Middle Eastern women. The incidence of pacifier use amongst breastfeeding women in Kuwait (40%) was approximately half that reported for women in Australia [43,44] and the USA [45], and introduction of a pacifier before four weeks of age was found to be negatively associated with any breastfeeding duration which is consistent with the international literature [46]. While the mechanism remains unclear, it has been suggested that the non-nutritive sucking on a pacifier reduces the frequency of nutritive sucking from the breast, thereby leading to less stimulation of the breast and consequently less milk production [43,45].

Finally, this study highlights the importance of social support for breastfeeding. Support can come from a woman’s partner, family and friends, and the degree to which each of these groups influences a woman’s decision to breastfeed varies according to the mother’s age, social class and cultural or ethnic background [47]. In traditional societies, women rely more on the advice and support of their mother, whereas in Western cultures they are more likely to identify their husband as their main source of support [48]. This and other studies of Muslim women have highlighted the importance of grandmothers both in providing practical support and as major influences on infant feeding decisions [8,49]. Advice received from their mother and mother-in-law can have both a negative and positive affect on a woman’s breastfeeding practices. For instance, on one hand, breastfeeding is promoted in the Quran (*Al Baqara*, *233*) and by elders as the desired way to feed an infant, and the mean duration of breastfeeding is longer in most Muslim countries than in Western countries. On the other hand, there is a common perception amongst older women that the heavier the baby the healthier he or she is. There is anecdotal evidence that Kuwaiti grandmothers often encourage topping up with formula to ensure the baby is satiated and to stop hunger cries, which explains in this study the positive association with any breastfeeding but not full breastfeeding.

This study also showed that a husband’s preference for breastfeeding over formula feeding was positively associated with breastfeeding initiation [30] and longer duration of full-breastfeeding which is consistent with Western studies [31,50,51]. To the best of our knowledge, no Middle-Eastern study has investigated previously the association of paternal attitudes and breastfeeding duration. There is, however, some evidence from Middle Eastern studies that support from a woman’s husband is important for breastfeeding success and a study of women in Saudi Arabia found that mothers were more likely to initiate breastfeeding if their partners supported breastfeeding [25]. A Turkish intervention study reported the positive effects of an antenatal education program for fathers on their reproductive health knowledge, attitudes and behaviours, and women whose husbands attended these classes reported that their husbands became more supportive and communicative [52].

Women everywhere doubt the adequacy of their milk supply [53] and in this study more than eight in 10 women gave this as one of their reasons for discontinuing breastfeeding. Perceived breast milk insufficiency or *insufficient milk syndrome* (IMS) is frequently associated with the premature introduction of complementary foods [9] and with the cessation of breastfeeding [14,16] in Middle Eastern countries. It has been proposed [53] that IMS is increasing with “aspects of ‘modernization’: urbanization, education, and female employment—factors that are repeatedly found to be inversely associated with both the prevalence and duration of breastfeeding” (p42). It has been suggested that ‘insufficient milk’ is given as a socially acceptable reason for discontinuing breastfeeding when a mother decides she no longer wishes to breastfeed [54] and that claims of IMS should not be taken literally when they occur in cultural contexts that present the use of infant formula as an acceptable, if not preferred, alternative [55].

As we have previously identified [30], there are a number of limitations to this study. Firstly, the sample size is relatively small and this is reflected in the wide confidence intervals around some of the adjusted hazard ratios reported. Secondly, we may have underestimated the rate of prelacteal feeding, which in this study was defined as within the first three days after birth. The average length of post-partum stay for Kuwaiti public hospitals is a maximum of two nights for uncomplicated deliveries and five nights for a caesarean section. Therefore, it is possible that some mothers discharged within 48 h may have gone on to supplement breastfeeding with formula following discharge from hospital and within this 72 h period. Given, however, that almost nine out of 10 infants received prelacteal feeding in hospital, any underestimation of prelacteal feeding is likely to have had only a negligible effect on the results. Finally, the number of women who delivered by caesarean section is three times that of the national average. While every attempt was made to recruit mothers within 72 h and in most cases 48 h, women who had undergone a caesarean section had a greater chance of being recruited because of their longer hospital stay.

The major strength of this study is that it was the first prospective study of infant feeding practices in Kuwait, all other previously reported studies being cross-sectional. Mother-infant dyads were followed from birth to 26 weeks with data being collected at five time points during this period, thus minimizing the potential for maternal recall bias [56] as women were recalling events close to the time at which they occurred. The findings of the study are consistent with those of other studies in the region and, in most instances, studies of Western women, and can be used to inform infant feeding policy, hospital practices and the design of breastfeeding promotion interventions.

## 5. Conclusions

The duration of breastfeeding amongst women in Kuwait, particularly Kuwaiti born women, appears to be declining. Exclusive breastfeeding is virtually non-existent with almost nine in 10 infants receiving prelacteal feeds within the first three days of birth. Full breastfeeding is also relatively uncommon, and by four weeks, less than one third of infants were fully breastfed, while at six months, only four in 10 infants were receiving some breast milk. This study identified a number of areas for intervention. Hospitals should follow the 10 Steps for Successful Breastfeeding [57] and, in particular, promote the early initiation of breastfeeding, encourage women to feed on demand and avoid the unnecessary practice of prelacteal feeding. Women and health professionals need to be alerted to the negative consequences of early pacifier use on breastfeeding duration. The role of family members should not be underestimated in planning breastfeeding interventions. Community-based interventions are needed to support women to breastfeed and to provide a supportive environment. Close family members, especially husbands and maternal grandmothers, should be targeted in these interventions to ensure higher rates of exclusive breastfeeding and prolonged duration.
